# GAME15: a pivotal nexus unlocking solanum alkaloid metabolism and biosynthesis underpinning the chemical defense and pharmacological potential in Solanum species

**DOI:** 10.3389/fpls.2025.1730777

**Published:** 2025-12-02

**Authors:** Xiaoxu Li, Ye Feng, Wencan He, Suxing Tuo, Guoxin Chen, Jinhao Sun, Zhiyuan Li, Kejun Zhong, Wei Luo, Bo Kong

**Affiliations:** 1 Technology Center, China Tobacco Hunan Industrial Co., Ltd., Changsha, China; 2 Beijing Life Science Academy, Beijing, China; 3 Tobacco Research Institute, Chinese Academy of Agricultural Sciences, Qingdao, China; 4 Technology Center, China Tobacco Jiangsu Industrial Co., Ltd., Nanjing, China

**Keywords:** GAME15, steroidal saponins and alkaloids, steroidal glycoalkaloids (SGAs), biosynthesis, chemical defense, Solanum

## 1 Introduction

The biosynthesis of steroidal glycoalkaloids (SGAs) and steroidal saponins (SSs) within the *Solanaceae* family serves as a fundamental basis for the plants’ defense mechanisms and pharmacological value (Zhao et al., 2021; Li and Luo, 2025). Despite extensive efforts to elucidate these pathways, attempts to reconstruct them in heterologous systems have consistently failed, indicating the presence of a critical “missing link.” Recent groundbreaking discoveries, particularly those surrounding the bifunctional cellulose synthase-like protein GAME15 (glycoalkaloid metabolism 15), have fundamentally altered our understanding of this field (Boccia et al., 2024; Lezin et al., 2025). GAME15 primarily acts as a non-classical scaffold protein in the endoplasmic reticulum, coordinating the formation of an efficient metabolic environment while also addressing challenges in recombinant efforts and highlighting the ecological role of steroidal saponins in plant defense (Boccia et al., 2024). Within the *Solanaceae* family, which includes economically significant crops such as tomatoes, potatoes, tobaccos, and eggplants, cholesterol-based steroid metabolites (particularly SGAs and SSs) play a central role in their chemical defenses and exhibit remarkable pharmacological properties (Gu et al., 2018; Akiyama et al., 2025; Li and Luo, 2025). SGAs are recognized as a primary defense mechanism against herbivores and pathogens due to their anti-nutritional and toxic effects. Conversely, SSs possess considerable potential in pharmaceutical and industrial applications, with compounds such as solamargine derived from black nightshade (*Solanum nigrum*) even being approved for the treatment of hepatocellular carcinoma (Lucier et al., 2024; Akiyama et al., 2025). Over the past few decades, significant progress has been made in identifying the enzymatic machinery involved in SGA biosynthesis, collectively referred to as GAME proteins. This group includes a range of cytochrome P450 enzymes (such as GAME6 and GAME8), dioxygenases (GAME11), and various transferases (Lezin et al., 2025). However, despite the identification of most catalytic genes, attempts to reconstruct the complete SGA biosynthetic pathway in heterologous hosts, such as *Nicotiana benthamiana*, have consistently encountered failures, strongly suggesting the existence of an elusive yet indispensable regulatory component (Boccia et al., 2024). Recent pioneering studies by Boccia et al. and Jozwiak et al., published in Science, reveal this long-sought “missing link”: the glycoalkaloid metabolism 15 (GAME15) protein (Boccia et al., 2024). Moreover, with GAME15 and late xylosylation defined, transient expression of 9–15 gene combinations in *Nicotiana benthamiana* reconstituted Solanum pathways and yielded approximately twenty steroidal end-products (Gharat et al., 2025). This article provides a perspective on how the discovery of GAME15 fundamentally reshapes our understanding of plant steroid metabolism, emphasizing its central role as a scaffold protein in forming metabolic bodies, which not only elucidates past challenges in recombinant efforts but also clarifies for the first time the ecological function of steroidal saponins in plant defense (Boccia et al., 2024; Jozwiak et al., 2024).

## 2 GAME15: a dual-function nexus of Solanum steroid metabolism

The discovery of GAME15 holds significant implications that extend far beyond the mere addition of an enzyme to metabolic pathways (Boccia et al., 2024; Jozwiak et al., 2024). Recent studies demonstrate that GAME15 functions both as an endoplasmic reticulum cholesterol glucuronosyltransferase and as a scaffold organizing early enzymes, thereby enabling flux to SGAs and steroidal saponins (Jozwiak et al., 2024; Boccia et al., 2024). More importantly, it unveils a complex regulatory mechanism that encompasses the intricate interplay between structural organization and catalytic activity. This revelation challenges the conventional perception of metabolic processes as linear sequences of reactions, suggesting that metabolic pathways may instead function as complex networks characterized by hierarchies and dynamic interactions (Boccia et al., 2024). Understanding this mechanism will offer new perspectives for advancements in metabolic engineering and biotechnology, thereby propelling further research and applications in related fields.

### 2.1 Architect of the metabolon: spatial organization for efficiency

The most profound revelation regarding GAME15 lies in its vital function as a scaffold protein that meticulously organizes the pivotal enzymes involved in the steroidal metabolic pathway on the membranes of the endoplasmic reticulum (Boccia et al., 2024). Experimental data, robustly supported by co-localization studies and protein-protein interaction assays, elucidate that GAME15 engages in direct interactions with upstream cholesterol biosynthesis enzymes, notably 7-dehydroxycholesterol reductase (7-DR2) and C5 sterol dehydrogenase (C5-SD2), as well as with downstream enzymes responsible for the critical process of cholesterol hydroxylation, including GAME6, GAME8, and GAME11 (Boccia et al., 2024). The spatial arrangement leads to the creation of a dynamic multi-enzyme complex known as a “metabolon,” which is crucial for enabling efficient biosynthesis and clarifying the challenges faced in previous heterologous reconstitution attempts. By fostering substrate channeling, the metabolon plays a crucial role in ensuring that metabolic intermediates are seamlessly transferred from the active site of one enzyme to that of another, which dramatically enhances local substrate concentrations and amplifies overall catalytic flux. Moreover, this channeling mechanism effectively reduces the likelihood of intermediate diffusion, thereby mitigating the risk of losses to competing biosynthetic pathways, such as phytosterol biosynthesis, or their potential toxic effects on cellular systems. The pronounced accumulation of cholesterol, coupled with the complete absence of SGAs and SSs in *GAME15* knockout mutants. These Evidences show that the indispensable role of GAME15 act as a central orchestrator of metabolic flux, underscoring its critical importance in maintaining homeostasis within the cellular milieu (Boccia et al., 2024). The conversion from unsaturated to saturated steroidal backbones begins with GAME25, and newer work completes terminal steps via RPG1/2 (Akiyama et al., 2025).

### 2.2 Unexpected catalytic activity and evolutionary repurposing

Intriguingly, beyond its scaffolding role, GAME15 also exhibits catalytic activity as a glucuronosyltransferase, a critical enzyme involved in the glucuronidation of cholesterol; this biochemical modification not only facilitates the subsequent hydroxylation reactions of cholesterol but also underscores the multifaceted nature of GAME15 (Boccia et al., 2024; Jozwiak et al., 2024; Kang and Wang, 2025). Such a dual functionality - serving as both an essential structural organizer and a catalytic enzyme - exemplifies the remarkable evolutionary ingenuity that characterizes the natural world (Jozwiak et al., 2024). The classification of GAME15 as a cellulose synthase-like (CSL) protein provides further insight into its functional repertoire. CSL proteins are traditionally recognized for their roles in primary metabolism, especially in the synthesis of cell walls, and the repurposing of a CSL protein like GAME15 for specialized metabolic processes illustrates a powerful evolutionary strategy employed by plants (Boccia et al., 2024; Jozwiak et al., 2024; Li et al., 2025). This strategy involves the co-option of pre-existing metabolic machinery to facilitate novel biochemical functions, thereby enhancing chemical diversity within the organism. Notably, this discovery builds upon previous studies that associated CSL proteins with triterpenoid saponin biosynthesis, mediated through glucuronidation. Nevertheless, in contrast to other CSLs with established glucuronosyltransferase activity, GAME15’s primary and arguably most critical function appears to reside in its role as a structural scaffold, emphasizing the intricate interplay between structure and function in plant biochemistry and the ongoing evolution of metabolic pathways (Boccia et al., 2024; Jozwiak et al., 2024).

## 3 Ecological revelation: unveiling the defensive role of steroidal saponins

While the defensive functions of SGAs have been well-documented within the field of phytochemistry, the ecological role of SSs in mediating plant defense, particularly within foliar structures, has remained an area of considerable uncertainty and limited investigation (Li et al., 2025). The recent discovery of a novel genetic locus known as GAME15 has provided a pivotal opportunity to systematically explore this under-researched aspect of plant defense mechanisms (Boccia et al., 2024; Jozwiak et al., 2024). Researchers developed *game15* knockout mutants in the species *Solanum nigrum*, resulting in plants that exhibit a complete deficiency in both SGAs and SSs (Boccia et al., 2024; Jozwiak et al., 2024). The resulting phenotypes of these knockout plants revealed a markedly increased susceptibility to a spectrum of insect herbivores, including the highly specialized black nightshade leafhopper (*Empoasca decipiens*), as well as the more generalized agricultural pest, the Colorado potato beetle (*Leptinotarsa decemlineata*) (Boccia et al., 2024; Jozwiak et al., 2024). Notably, because the leaves of *Solanum nigrum* predominantly accumulate steroidal saponins and contain little to no SGAs, these findings provide direct evidence that saponins are key contributors to leaf-based plant defense. This research not only addresses a critical gap in our understanding of plant defense theory but also paves the way for innovative agricultural practices aimed at developing pest-resistant crop varieties, thus contributing to sustainable agricultural systems and food security at large.

## 4 Discussion

The discovery of GAME15 signifies a momentous achievement in the realm of plant specialized metabolism, bridging a crucial gap in our understanding of intricate biosynthetic pathways and substantially enhancing our comprehension of how metabolons regulate the flow of metabolic processes (Boccia et al., 2024; Jozwiak et al., 2024). By incorporating *GAME15* into heterologous expression systems, such as yeast and *N. benthamiana*, researchers can now facilitate the efficient and scalable production of valuable compounds, including steroidal saponins like uttroside B and specific glycoalkaloids (Boccia et al., 2024; Jozwiak et al., 2024). This newfound capability not only paves the way for the development of optimized “cell factories” aimed at sustainable biomanufacturing of pharmaceutical precursors and other bioactive substances but also underscores the potential for significant advancements in crop improvement endeavors (Boccia et al., 2024; Jozwiak et al., 2024). In this context, *GAME15* and the steroidal saponin pathway it orchestrates emerge as novel targets for enhancing plant resilience, whereby precision gene-editing techniques can be applied to meticulously fine-tune the accumulation of defensive saponins in edible plant tissues. Such advancements promise to bolster natural pest resistance while simultaneously mitigating the dependence on chemical pesticides (Boccia et al., 2024; Jozwiak et al., 2024; Lezin et al., 2025). Additionally, a profound understanding of this biosynthetic pathway empowers researchers to accurately reduce the presence of anti-nutritional saponins in crops intended for human consumption, effectively balancing the dual imperatives of plant defense and food safety. However, despite these substantial advancements, several pressing questions remain unresolved. The intricate structural basis underlying GAME15’s dual functionality, particularly its interactions with and organization of client enzymes, has yet to be fully elucidated. Furthermore, a comprehensive understanding of the upstream regulatory networks governing GAME15’s activity and the dynamics of metabolon formation is vital for optimizing metabolic flux within this specialized metabolic framework (Boccia et al., 2024; Jozwiak et al., 2024; Lucier et al., 2024). Additionally, the enzymatic mechanism through which the glucuronic acid moiety, introduced by GAME15, is later excised within the pathway warrants further inquiry. Addressing these scientific enigmas will not only enhance our grasp of this complex metabolic network but also unlock even greater potential for targeted metabolic engineering initiatives (Boccia et al., 2024; Jozwiak et al., 2024; Kang and Wang, 2025). In summary, the discovery of GAME15 exemplifies how basic science fuels applied innovation: it solves a long-standing metabolic puzzle and provides researchers with powerful new tools to tap nature’s chemical diversity, enabling practical solutions to global challenges in agriculture and healthcare.
